# 10H-phenothiazine exerts beneficial effects in spinal muscular atrophy in vitro and in vivo models

**DOI:** 10.1038/s41598-025-28547-9

**Published:** 2025-12-16

**Authors:** Daniela Maria Rasà, Serena Stanga, Pamela Santonicola, Nadia Mazzarella, Antonella Camera, Ilenia Matino, Giuseppina Zampi, Marina Boido, Elia Di Schiavi, Alessandro Vercelli

**Affiliations:** 1 Neuroscience Institute Cavalieri Ottolenghi, Department of Neuroscience “Rita Levi Montalcini”, University of Turin, Turin, Italy; 2 Institute of Biosciences and BioResources (IBBR), National Research Council (CNR), Naples, Italy; 3 Institute of Genetics and Biophysics “Adriano Buzzati-Traverso” (IGB-ABT), National Research Council (CNR), Naples, Italy

**Keywords:** Disease-modifying agents, Morphometric analyses, Motor neuron disease, SMN-independent strategies, Drug repurposing, Cell biology, Neuroscience

## Abstract

Spinal Muscular Atrophy (SMA) is a neurodegenerative disorder affecting lower motor neurons (MNs) and leading to muscle atrophy, due to mutation of the *SMN1* gene, which encodes SMN protein. Experimental studies also demonstrated the upper MN impairment. The available approved drugs for SMA increase the SMN protein production. Although effective, outcomes are dependent upon treatment timing and disease severity. Drug repositioning may represent a valid strategy to identify new treatments by repurposing FDA/EMA-approved drugs that, combined with the available ones, could delay neurodegeneration. To this aim, for the first time we used primary cortical neurons derived from the SMNΔ7 mice as defective in vitro disease model, to preliminary assess drug efficacy on neuronal survival and morphology. Under basal conditions, SMA cortical neurons showed significantly reduced vitality and altered morphology compared to WT neurons. All the parameters were rescued after treatment with known compounds (Valproic Acid, 4-aminopyridine and N-acetylcysteine), already tested in either preclinical or clinical context for SMA. We then investigated for the first time in SMA pathology the efficacy of 10H-phenothiazine (10H-PTZ), known to exert neuroprotection and to target altered mechanisms in Parkinson’s and Alzheimer’s disease. Its administration to SMA cortical neurons induced significant protective effects on both neuronal survival and morphology that were further confirmed in vivo, in a *C. elegans* SMA model. Overall, our results provide valuable insights, both in vitro and in vivo, into the potential of 10 H-PTZ repurposing for SMA, although additional functional studies will be required.

**Supplementary Information:**

The online version contains supplementary material available at 10.1038/s41598-025-28547-9.

## Introduction

Spinal Muscular Atrophy (SMA) is a genetic disorder characterized by the progressive degeneration of lower motor neurons (MNs), leading to muscle wasting and weakness. Interestingly, a selective decrease in the number of layer V pyramidal neurons (upper MNs) in the motor cortex has been also reported^1^, making the SMA pathogenesis more complex than previously described. SMA is primarily caused by mutations or deletions in the Survival of Motor Neuron 1 (*SMN1*) gene, which results in insufficient levels of the SMN protein, crucial for MN survival and function^2^. This autosomal recessive disease presents with various forms, from severe (Type I) to mild (Type IV), depending on the extent of SMN protein deficiency and the presence of the *SMN2* gene, a nearly identical copy of *SMN1* that produces a limited amount of functional SMN protein^3^. Current therapeutic approaches for SMA aim to increase the production of functional SMN protein. The first FDA-approved treatment, Nusinersen (Spinraza), is an antisense oligonucleotide that modulates the splicing of SMN2 pre-mRNA to increase the production of full-length SMN protein^4^. Another therapy is Onasemnogene abeparvovec (Zolgensma), a gene therapy that delivers a functional copy of the *SMN1* gene via an adeno-associated virus vector, effectively restoring SMN protein levels^5^. Additionally, Risdiplam (Evrysdi) is an orally administered small molecule that promotes *SMN2* exon 7 inclusion, enhancing SMN protein synthesis^6^.

Although highly effective when early administered, the efficacy of the currently approved therapeutic approaches drastically decreases when administered in advanced stages of the disease^7^. Moreover, the three available “disease-modifying therapies” do not assure a tunable SMN production, as occurs in physiological conditions^8^, and in some cases could even triggers SMA-like pathogenic events through toxic gain of function mechanism^9^. Therefore, identifying combinatorial treatments that, in association with the SMN-dependent therapies, could delay MNs’ degeneration by targeting other molecular pathways, such as oxidative stress, apoptosis, mitochondrial dynamics, known to be altered in SMA^10^, may represent a pivotal therapeutic strategy for those patients who currently poorly benefit from Spinraza, Zolgensma or Evrysdi.

In light of this, to develop a complementary approach, we followed a drug repositioning (DR) strategy. This method leverages the known safety profiles, pharmacokinetics and manufacturing processes of established medications, thereby reducing the time, cost, and risk associated with drug development. By identifying novel applications for drugs that are already FDA/EMA-approved or in clinical use, we aim to accelerate the development of complementary treatments for patients.

Therefore, following this strategy and selecting drugs of potential interest, we tested a known small molecule, 10H-phenothiazine (10H-PTZ, which derivatives are FDA approved), which has never been previously tested in experimental and clinical contexts for SMA. 10H-PTZ and its derivatives are commonly administered as antipsychotics, antiviral and analgesic agents^11^. However, 10H-PTZ itself^12^ or complexed with other molecules (such as 10-carboxamide or Donepezil) also showed neuroprotective effects in Parkinson’s disease (PD)^13,14^ and Alzheimer’s disease (AD), also exerting brain protection from oxidative stress^15–17^. Therefore, they might be of potential interest for SMA.

First, we tested 10H-PTZ in primary cortical neurons derived from the SMNΔ7 mice, a severe experimental SMA model. To the best of our knowledge, we used for the first time SMA-derived primary cortical neurons as a defective in vitro disease model, demonstrating its reliability for preliminary drug efficacy screening for cells viability and morphology. To further validate the cell model, we used three compounds known to exert positive effects in SMA context: Valproic acid (VPA), evaluated in clinical trial, alone or in combination with Carnitine or L-Carnitine – NCT01033331, NCT00481013, NCT00374075, NCT00661453, NCT00227266, NCT01671384; https://www.clinicaltrials.gov/)^18,19^; 4-aminopyridine (4-AP; undergone in clinical trial – NCT01645787 – https://www.clinicaltrials.gov/ - and studied in different SMA experimental works)^20–22^; and N-acetylcysteine (NAC; evaluated in preclinical SMA studies, but never in clinical trials)^23,24^.

Finally, the 10H-PTZ has been evaluated in an in vivo model, *Caenorhabditis elegans (C. elegans)*, a small model system that preserves in a whole-animal context intact cell-to-cell communications and many aspects of the animals’ metabolism, ideal for preliminary drug validation.

Thanks to this work, we confirmed the reliability of cortical neuron culture as a valid SMA in vitro model for screening therapeutic treatments and we demonstrated the potential of 10H-PTZ for drug repurposing for SMA. Further studies, including direct functional assays in mammalian models, will be necessary to fully support 10H-PTZ use for SMA patients.

## Results

### Primary cortical neurons of SMA mice show a defective neuronal morphology

**Fig. 1:**
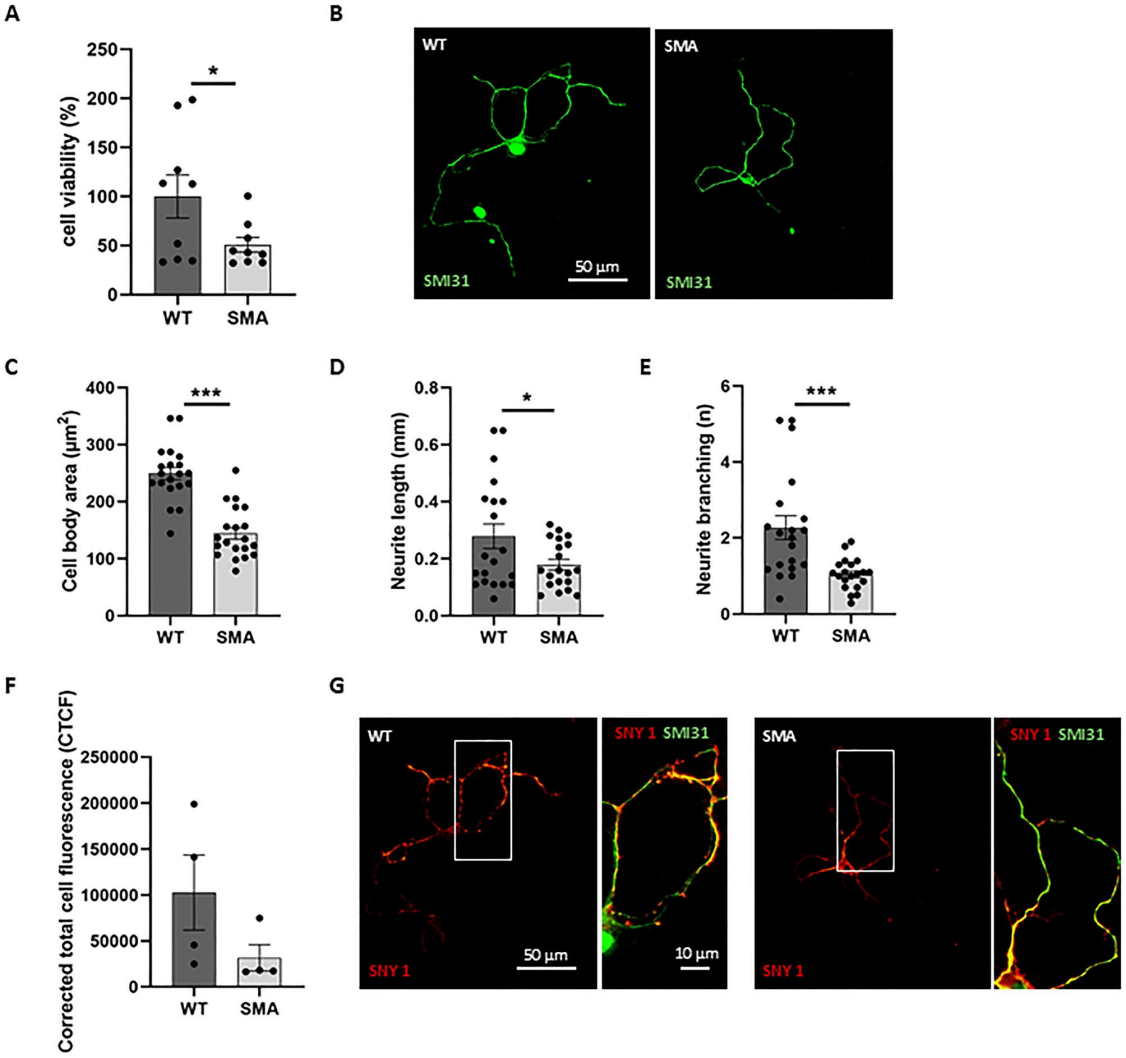
Primary cortical neurons of SMA mice show a defective phenotype. (**A**) Cell viability was evaluated in WT and SMA primary cortical neurons by MTT assay. Results are represented as mean ± SD of three independent experiments. Unpaired Student t-test SMA vs. WT *p < 0.05. (**B**) Primary cultures of WT and SMA cortical neurons immunolabeled with anti-SMI31 antibody (green). Scale bar = 50 μm. (**C**–**E**) WT e SMA morphological parameters were measured using Neurolucida and NeuroExplorer software. Results are represented as mean ± SD of five independent experiments (*n* ≥ 10 neurons for each experiment). Unpaired Student t-test SMA vs. WT *p < 0.05, ***p < 0.001. (**F**) Quantification of synapsin signal as the corrected total cell fluorescence (CTCF) shows a trend to the reduction in SMA neurons compared to WT. (**G**) Primary cultures of WT and SMA cortical neurons were immunolabeled by anti-synapsin antibody (red). A magnification of the cropped area (indicated by a white rectangle) is also shown, displaying the merged signals of the anti-SMI31 antibody (green) and the anti-synapsin 1 antibody (red). The orange/yellow signal represents the colocalization of the two markers. Scale bar = 50 μm. Magnification: scale bar = 10 μm.

First, we studied the phenotype of primary cortical neurons derived from newborn wild-type (WT) and SMA mice in vitro. At day in vitro (div) 7, neurons were mature, and neurite branching and complexity were evident. At this time point, we first evaluated the cell viability by MTT assay in basal conditions: we observed a reduced survival for SMA neurons compared to WT, as sign of the expected neurodegeneration due to the lack of SMN protein (Fig. 1A).

To assess cell morphological parameters such as cell body area, length and branching of neuronal processes, cortical neurons have been immunostained with anti-SMI31 antibody, a neuronal marker labeling cell axons, dendrites and soma (Fig. 1B). The analysis of morphological parameters revealed that the cell body area, and the neurite length and branching of SMA cortical neurons were significantly reduced compared to WT (Fig. 1C-E). Furthermore, we evaluated the signal intensity and distribution of synapsin in SMA compared to WT cultures, since the synaptic vesicle distribution can be a noteworthy indicator of cell maturation and complexity of neuronal network^25^. We did not observe statistically significant differences in the synapsin corrected total cell fluorescence (CTCF) between WT and SMA (Fig. 1F); however, interestingly, we observed a different distribution of the fluorescent signal, with synapsin retained close to the cellular bodies in SMA neurons, while uniformly distributed all along neuronal processes in WT cells (Fig. 1G). Since synapsin is involved in neuronal development, synaptogenesis and in the modulation of neurotransmitters’ release, its absence at neuronal active zones could support the incomplete neuronal maturation observed above. Moreover, its role in synaptic vesicle dynamics could possibly impact the functionality of the neuronal network^26^.

**Fig. 2:**
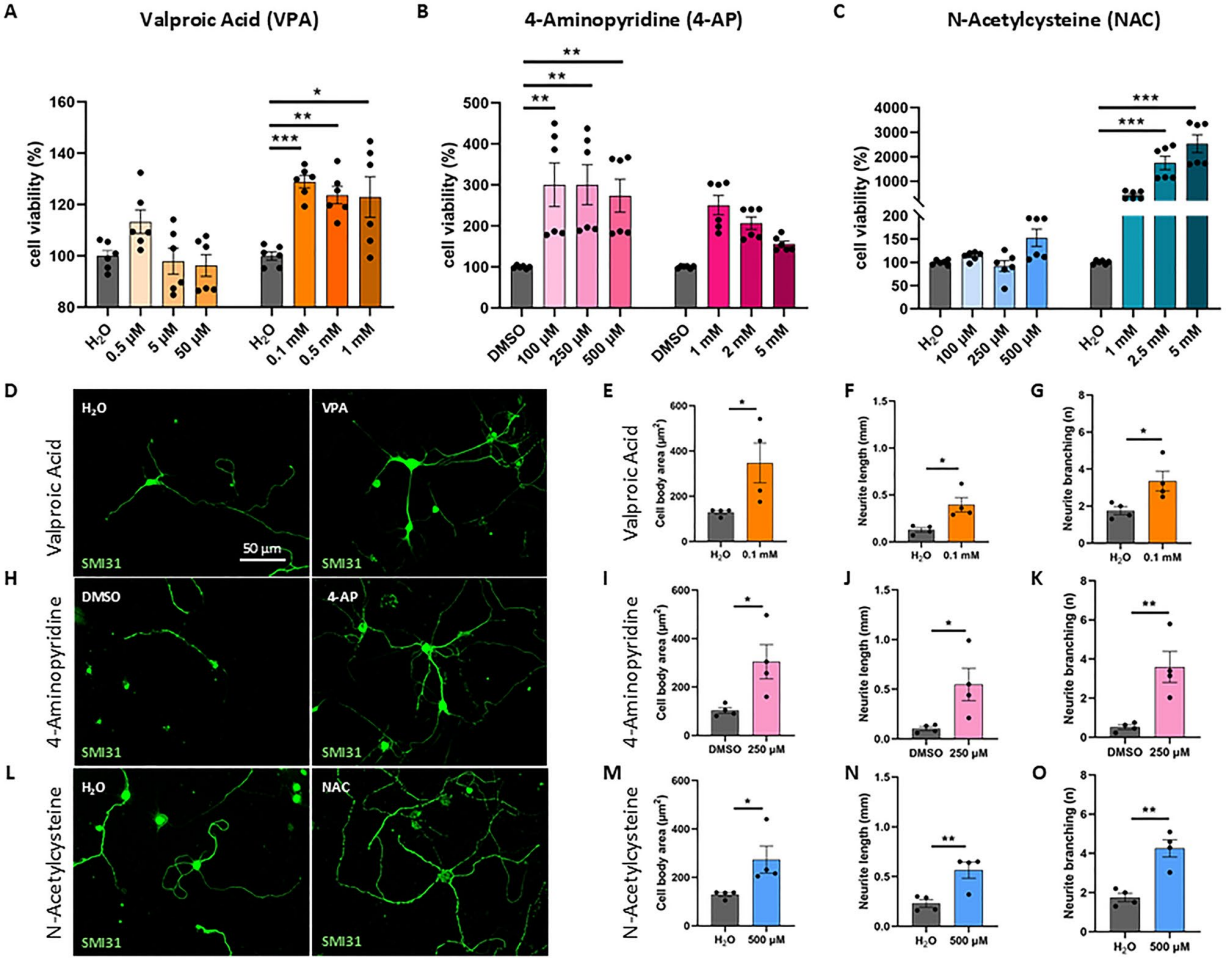
Cell viability and morphological analysis after treatment with positive controls (VPA, 4-AP and NAC). (**A**–**C**) SMA primary cortical neurons were treated with different concentrations of each compound: the cell viability was evaluated after 48 h by MTT assay, and compared to SMA neurons with the vehicle. Results are represented as the mean ± SD. One-way ANOVA followed by Tukey’s multiple comparison test: treated SMA vs. vehicle *p < 0.05, **p < 0.01 and ***<0.001. (**D**,**H**,**L**) SMA primary cortical neurons were treated and immunolabeled by anti-SMI31 antibody (green). Scale bar = 50 μm. (**E**–**G**, **I**–**K**, **M**–**O**) Morphological parameters of treated SMA cells measured using Neurolucida and NeuroExplorer software. Results are represented as mean ± SD of four independent experiments (*n* ≥ 10 neurons for each experiment). Student t-test: treated SMA vs. vehicle *p < 0.05, **p < 0.01.

Altogether, our results indicate that primary cortical neurons from SMA mice show evident phenotypic defects compared to WT cells, supporting their use as a reliable in vitro model for a preliminary therapeutic screening in SMA. Once validated viability and morphological differences between SMA and WT cortical neurons, we studied a possible rescue of disease-related impairments using three drugs already known to be effective in SMA models, serving as positive controls: VPA, 4-AP and NAC^18,22,23^. Each drug was administered at different concentrations to SMA cortical neurons, to exclude toxic doses (Table 1). The cell viability was measured by MTT assay, as shown in Fig. 2A-C. The results showed that for all the controls, none of the tested doses was toxic. Moreover, we observed that the higher concentrations of VPA and NAC (respectively 0.1mM-0.5mM-1mM, and 2.5mM-5mM) (Fig. 2A-C), and the lower concentrations of 4-AP (100 µM–250 µM–500 µM) (Fig. 2B) were able to significantly enhance cell viability. However, for NAC, we observed an unusually rapid formation of formazan crystals during the reaction, which we interpreted as a potential false positive in the MTT assay.

**Table 1:** List and concentrations of the compounds tested for 3-[4,5-dimethylthiazol-2-yl]-2,5 Diphenyl tetrazolium bromide (MTT) assay and immunofluorescence.

Compound	Concentration for MTT	Concentration for IF	Solvent
Valproic acid (VPA)	0.5 µM, 5 µM, 50 µM, 0.1mM, 0.5mM, 1mM	0.1mM, 1mM	H_2_O
4-aminopyridine (4-AP)	100 µM, 250 µM, 500 µM, 1mM, 2mM, 5mM	250 µM, 2mM	DMSO
N-acetylcysteine (NAC)	100 µM, 250 µM, 500 µM, 1mM, 2.5mM, 5mM	500 µM, 5mM	H_2_O
10H-phenothiazine (10H-PTZ)	1nM, 10nM, 100nM, 10 µM, 20 µM, 50 µM	10nM, 20 µM	DMSO

Based on these findings, we also studied some neuronal morphological parameters (soma size, neurite length and branching) after 48 h of treatment, using the best concentrations of VPA, 4-AP and NAC that resulted significantly active in the MTT assay (0.1 mM, 250 µM and 5 mM, respectively). As expected, both 0.1mM VPA and 250µM 4-AP significantly improved all the morphological parameters analyzed compared to vehicle (VHL)-treated SMA cells (Fig. 2D-K). Concerning NAC, we did not observe relevant differences with the 5mM concentration (Fig. S1), probably due to the MTT assay results above-mentioned. However, by testing a lower NAC concentration (500 µM), all the morphological parameters analyzed were significantly increased, compared with to VHL-treated SMA cells (Fig. 2L-O).

### Evaluation of the efficacy of the candidate compound in rescuing viability and morphology of SMA cortical neurons

**Fig. 3:**
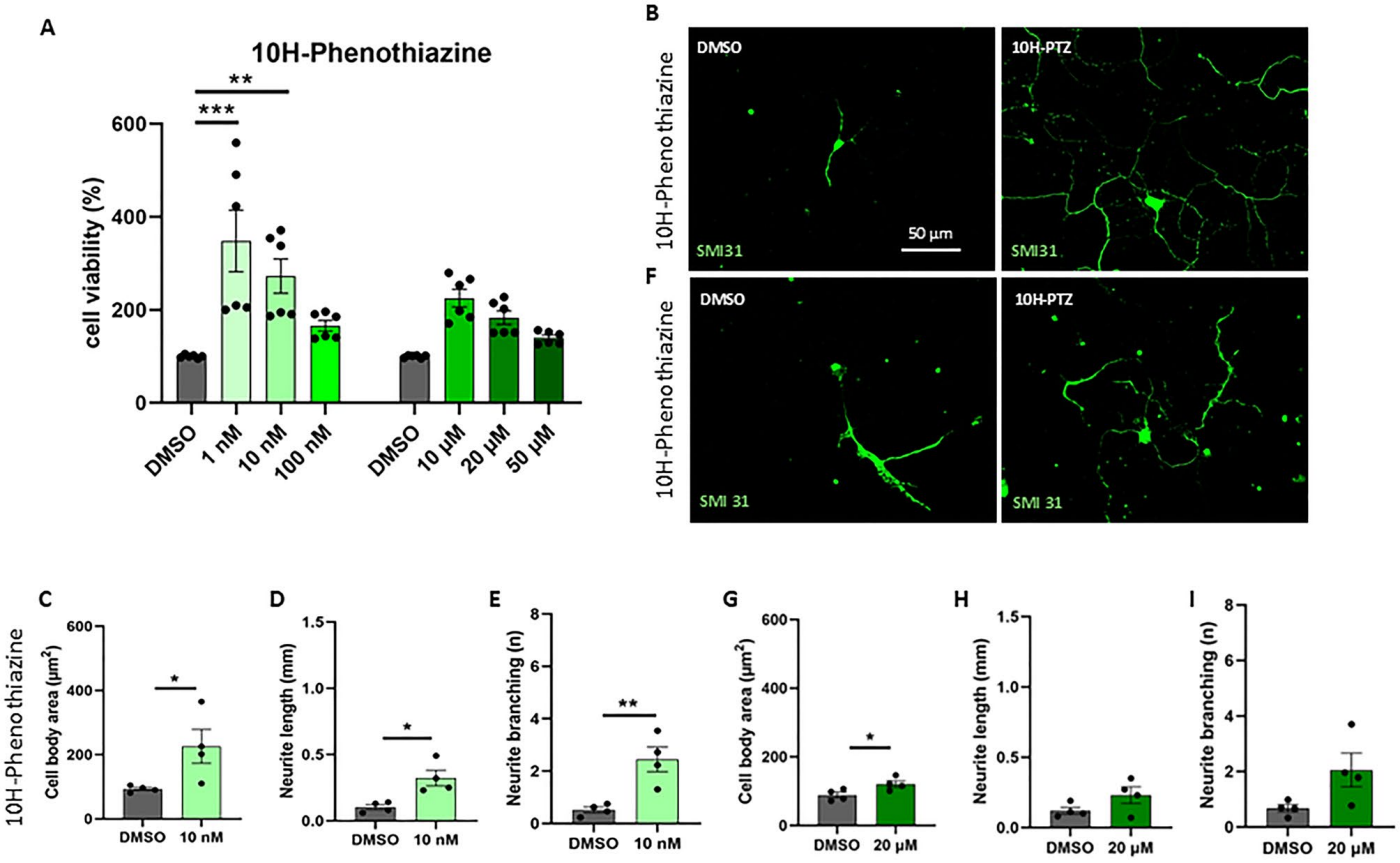
Cell viability and morphological analysis after 10H-PTZ treatment. (**A**) SMA primary cortical neurons were treated with different compound concentrations: the cell viability was evaluated after 48 h by MTT assay, and compared to SMA neurons with the vehicle. Results are represented as the mean ± SD. One-way ANOVA followed by Tukey’s multiple comparison test: treated SMA vs. vehicle *p < 0.05, **p < 0.01 and ***<0.001. (**B**,** F**) SMA primary cortical neurons were treated and immunolabeled by anti-SMI31 antibody (green). Scale bar = 50 μm. (**C**–**E**, **G–I**) Morphological parameters of treated SMA cells measured using Neurolucida and NeuroExplorer software. Results are represented as mean ± SD of four independent experiments (*n* ≥ 10 neurons for each experiment). Student t-test: treated SMA vs. vehicle *p < 0.05, **p < 0.01.

After validating the use of cortical neuron cultures as a valid model for testing drug efficacy, we treated the cells with the candidate molecule: 10H-PTZ. Different concentrations of the compound were administered to SMA cortical neurons in order to determine their possible toxicity (Table 1). The MTT results showed that all the concentrations tested were not toxic (Fig. 3A) and the lower concentrations of 10H-PTZ (1nM and 10nM) were able to significantly support cell viability.

We then selected both a low and a high concentration, and we analyzed the morphological parameters of treated cells for 48 h: interestingly, the lowest concentration (10nM) was able to improve the general phenotype of SMA cortical neurons, significantly increasing cell body area, neurite length and branching compared to VHL-treated SMA cells (Fig. 3B–E). Instead, 20 µM 10H-PTZ showed only a significant effect on the cell body area compared to VHL-treated SMA neurons (Fig. 3F–I).

### Synaptic vesicle distribution is affected by candidate compound treatment

**Fig. 4:**
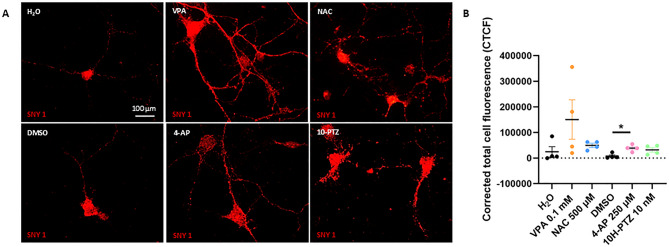
Synapsin distribution after treatments. (**A**) Images of the synapsin vesicle distribution in SMA treated vs. vehicle neurons. Vehicle for VPA and NAC is H_2_O; vehicle for 10H-PTZ and 4-AP is DMSO. Scale bar = 100 μm. (**B**) Quantification of the corrected total cell fluorescence (CTCF) for all the compounds. Results are represented as mean ± SD of four independent experiments (*n* ≥ 10 neurons for each experiment). One way ANOVA, Dunnet Post Hoc Test treated SMA vs. vehicle *p < 0.05.

To further assess the effects of the most effective concentrations, for each candidate and control drug we analyzed the synapsin-positive vesicles distribution and the CTCF. All the tested drugs were able to rescue a uniform vesicle distribution throughout the entire cell (Fig. 4A). Moreover, by analyzing the CTCF, we observed a significant increase of synapsin signal in SMA treated cortical neurons compared to VHL SMA neurons only after treatment 4-AP (Fig. 4B). This indicates that the treatment is stimulating the expression of one of the major integral membrane proteins of synaptic vesicles, synapsin 1, involved in neurotransmitters’ release.

### Neuroprotective effects of candidate compounds in a *C. elegans* model of SMA

**Fig. 5:**
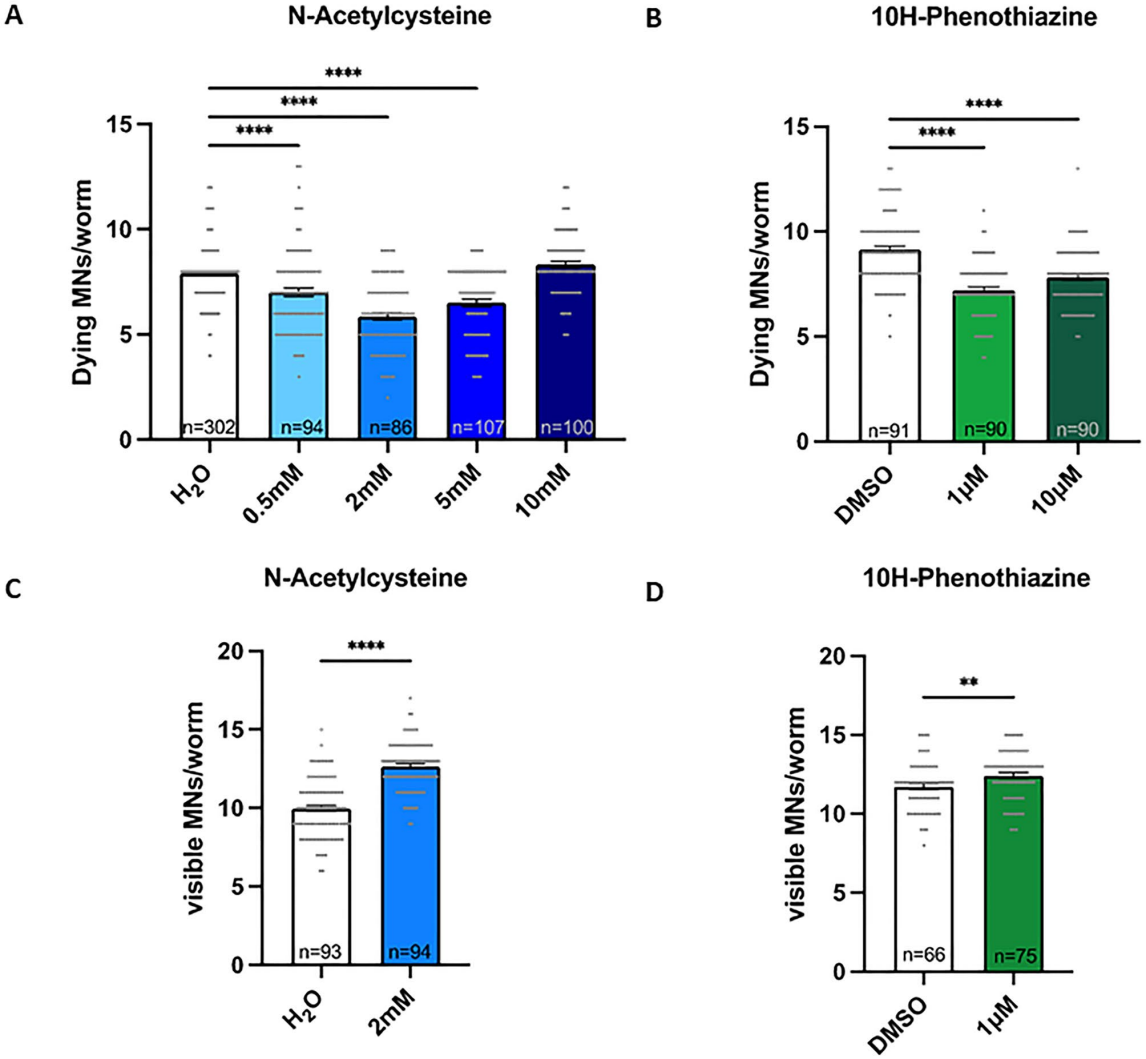
In vivo effect of neuroprotective molecules on a *C. elegans* model of SMA. (**A**) Quantification of the number of dying MNs per animal after treatment with vehicle (H_2_O) and increasing concentrations of NAC 0.5, 2, 5 and 10 mM. (**B**) Quantification of the number of dying MNs per animal after treatment with vehicle (DMSO 1%) and increasing concentrations of 10H-PTZ 1 and 10 µM. One-way ANOVA Kruskal-Wallis Dunn’s multiple comparison test ****p < 0.0001. A and B. Bar represents the mean and each dot corresponds to the number of dying MNs scored in a worm. (**C**) Quantification of the number of visible MNs expressing GFP per animal after treatment with vehicle (H_2_O) and NAC 2 mM. (**D**) Quantification of the number of visible MNs expressing GFP per animal after treatment with vehicle (DMSO 1%) and 10H-PTZ 1 µM. Mann Whitney *t-*test ****p < 0.0001; **p < 0.05. C and D. Bar represents the mean and each dot corresponds to the number of visible MNs expressing GFP scored in a worm. n is the number of animals analysed in all panels.

Finally, to validate also in vivo the effect of 10H-PTZ, we exploited the *C. elegans* animal model. To specifically assess its neuroprotective effect in the SMA context, we used the transgenic model where *smn-1* is specifically silenced only in 19 D-type MNs^27^, thus avoiding the pleiotropic effect of *smn-1* depletion in all the organism (that on the contrary occurs in SMA patients). In this model, it is possible to evaluate the apoptotic death of MNs in living animals by using a fluorescent cell death marker. As positive control drug, we employed NAC. By testing different concentrations of NAC and 10H-phenothiazine, we observed a dose-dependent effect in rescuing MN death of NAC with the lower concentrations tested (0.5, 2 and 5 mM) (Fig. 5A) and with both concentrations of 10H-phenothiazine (1 and 10 µM) (Fig. 5B). Moreover, in order to follow the initial stages of MN loss, we studied in *C. elegans* the early events of neurodegeneration by monitoring the number of visible/viable MNs using double transgenic animals that also express the GFP in the same MNs^27^. This phenotype has been demonstrated to precede the appearance of the apoptotic marker. Therefore, we choose the most effective doses of NAC and 10H-phenothiazine to test their efficacy also on the earliest neurodegenerative phenotype. Interestingly, we observed a significantly higher number of visible/viable MNs after treatment with both molecules (Fig. 5C, D).

Thus, we can conclude that 10H-PTZ exerts a neuroprotective effect in a SMA *C. elegans* models, consistent with the other beneficial effects obtained on cortical neurons.

## Discussion

Currently, it is evident that the approved therapeutic approaches for SMA treatment might not address the global neurodegenerative process that causes progressive functional decline beyond childhood in less severe SMA types^28^. Patients treated with actual SMN-based therapies might simply present delayed instead of rescued symptoms, if recovery of the neuromuscular system is incomplete. Moreover, a large number of older patients living with chronic symptoms might not benefit from SMN-inducing treatments. Additionally, they do not allow precise, tunable control of SMN expression and might even trigger SMA-like pathogenic events through toxic gain of function mechanism^9^. Finally, the long-term effects are still unknown: indeed, it has been demonstrated that a prolonged AAV9-mediated SMN overexpression in mice induces dose-dependent, late-onset motor dysfunctions associated with neurodegeneration^9^.

In this framework, the drug repurposing (DR) strategy appears an interesting approach to provide alternative therapeutic options, potentially targeting SMN-independent mechanisms. Moreover, compared to *de novo* drug development, DR minimizes the time and cost of drug commercialization^29^. Indeed, the development of new molecules usually involves several phases that may require up to 10–17 years and it requires preclinical and clinical phases I and II^30^. The reduction in time and costs of DR represents a great advantage and makes DR an increasingly used approach in neurodegenerative diseases, including SMA^31,32^. Our work aims to address this challenge, by providing a new experimental in vitro model applied for the first time in the context of DR strategies for SMA field.

We have previously demonstrated that neuronal degeneration in SMA involves cortical regions of SMNΔ7 mice^1^. Therefore, we cultured primary cortical neurons as a novel cellular model to study neurodegeneration in SMA and to serve as a platform for testing drug efficacy. Indeed, primary cortical neurons represent a long-established in vitro setting to study neuronal maturation for many different models of neurodegenerative disease^33,34^.

Interestingly, we observed that in terms of cell viability, morphological parameters and synaptic vesicle distribution there is a significant impairment in cortical neurons from SMA mice compared to WT cells. The impaired phenotype has been rescued after treatment with compounds already known to be active on SMA and used here as positive controls (VPA, 4-AP and NAC). Although SMA is commonly reported to affect the lower MNs, our data, together with several other studies showing the lethal consequences of *SMN1* loss on the whole organism, including patient’s brain^35–37^, support the currently established concept that SMA is a multisystemic disease^38^.

We show here that the candidate molecule, 10H-PTZ, was capable to rescue the morphological defects of SMA primary cortical neuron cultures. These results, for the first time associated to SMA pathology, are consistent with the existing literature of the positive effect of 10H-PTZ treatment for different neurodegenerative diseases^39^. In addition, 10H-PTZ had a positive effect on the synaptic vesicle distribution in our in vitro model for SMA. Although CTCF quantification only showed significant results in cells treated with 4-AP (used as positive control), we observed a redistribution of synaptic vesicles to neurites in cells treated with the candidate compound indicating an increased availability at the synaptic terminals with possible consequences in neuronal activity^40–44^. However, additional investigation to confirm the improved functional activity would be important to fully capture the efficacy of the pharmacological treatment. Concerning NAC (the only among the control compounds that did not reach yet the clinical trial stage for SMA), we confirmed its neuroprotective effect in SMA cultured cortical neurons, extending the previous findings in the context of other neurodegenerative and MN diseases^23,24,45^.

To further investigate the effects of 10H-PTZ in vivo in a whole organism, we tested it in a *C. elegans* model of SMA, using NAC as positive control. Being an invertebrate, *C. elegans* implements the principles of replacement, reduction and refinement (3Rs), thus being a first screening step prior to the use of in vivo mammalian models on much more restricted questions. The model organism *C. elegans* has been very helpful in elucidating some of the molecular mechanisms underlying SMA. The simplicity of its nervous system, coupled with well-characterized genetics, makes it an ideal system for studying neuromuscular diseases. Notably, *C. elegans* possesses a homolog of the human SMN gene, known as *smn-1*, which is essential for motor neuron function and animal viability^46–48^. Additionally, knock-out or hypomorphic alleles of the *smn-1* gene have facilitated the identification of genetic and chemical modifiers of SMA phenotypes^49^. We developed transgenic animals silenced in *smn-1* in a subclass of 19 D-type motor neurons (MNs) that present some key features of SMA, in particular, impaired locomotion, neuronal degeneration and neuronal death^27^. Although this model does not fully recapitulate the human disease (where the SMN lack affects multiple tissues and cell types, not just the MNs), it has the advantage to discriminate the specific neuroprotective effect of 10H-PTZ on MNs. These animals are viable and fertile, so we largely used them to identify SMA genetic modifiers through candidate^50–53^ and unbiased genetic screenings^54,55^, or to find modifying compounds that partially prevent neuronal death^56^, including valproic acid, which was successfully used in other models of SMA^18,27,57^. Interestingly, 10H-PTZ showed the capability to significantly increase the number of visible MNs after treatment in the *C. elegans* model of SMA, to an extent comparable to that observed with NAC. Functional tests to measure how 10H-PTZ affects biological and physiological processes in mammalian models for SMA are essential to support its use for SMA patients. Further studies in mammalian models to determine absorption, distribution, metabolism, excretion aspects, and the drug specific pharmacokinetics/pharmacodynamics, would help the transition to identify a relevant dose for humans.

Altogether, these findings may represent valuable insight into the potential neuroprotective role of 10H-PTZ in SMA. Moreover, this work confirms that cortical neuron cultures represent a reliable in vitro SMA model for drug testing.

## Materials and methods

### Mouse model and genotyping

The SMNΔ7 mice (Stock No. 005025, The Jackson Laboratory https://www.jax.org/strain/005025), which recapitulate a severe model of SMA, are homozygous for both human transgenes h*SMN*2 and *SMNΔ7*, so that mice that are also homozygous null for mouse *Smn* (*Smn*^−/−^) survive to the end of the second postnatal week. Heterozygous mice for S*mn* deletion (*Smn*^*+/−*^, carrier) are fully viable and their interbreeding is required to maintain the colony. The pups were genotyped by PCR assays to assess the presence of two human transgenes and the three possible genotype variants of the mouse *Smn* locus (*Smn*^+/+^, *Smn*^−/−^ and *Smn*^+/−^)^58^. Data were obtained from knock-out SMA (*Smn*^*−/*^, SMA) and wild type (*Smn*^*+/+*^, WT) mice. Both male and female mice were used for the following experiments and were sacrificed at postnatal day 1 (P1).

All the experimental procedures were performed in strict accordance with institutional guidelines in compliance with national (D.L. N.26, 04/03/2014) and international laws, rules and policies (new directive 2010/63/EU). In addition, the experimental procedures were also in accordance with ARRIVE guidelines. The study was approved by the Italian Ministry of Health (protocol #160/2020-PR). Additionally, the ad hoc Ethical Committee of the University of Turin approved this study. All efforts were made to minimize the number of animals used and their suffering.

### Primary neuronal cultures

Primary cultures of cortical neurons were prepared from P1 WT and SMA newborns, as previously described^59^. Briefly, the pups were deeply anesthetized by hypothermia: they were then rapidly decapitated, and their brains were collected. Cortices were dissected in Neurobasal medium supplemented with 2% v/v B-27 medium, with 0.5 mM L-glutamine and with penicillin–streptomycin (50 mg/ml of each) and immediately digested with a trypsin solution (220 unit/mg) containing DNAse (1 mg/ml) at 37 °C for 3 min. After removal of the trypsin/DNAse solution, cortices were further dissociated in Neurobasal medium supplemented with DNAse (0.5 mg/ml). Cells were plated in culture dishes pre-treated with 10 µg/ml poly-L-lysine in PBS. Before each treatment, cells were cultured for 5 days in vitro in Neurobasal medium supplemented with 2% v/v B-27 medium, with 0.5 mM L-glutamine and with penicillin–streptomycin (50 mg/ml of each) and were maintained at 37 °C in a 5% CO2 atmosphere. Under these conditions, neuronal cells display high differentiation (in terms of neurite development and complexity) and survival rate. At day in vitro 5 (div5) neurons have been treated for 48 h with 3 positive controls (VPA, 4-AP and NAC) or a candidate compound (10H-PTZ) at different concentrations, as shown below (Table 1) and analyzed at div7 for the following analyses. Indeed, at this time point, neurons are mature and neuronal networks are clearly established. Each condition has been compared to non-treated cells with the respective vehicles (solvent) as follows: H_2_O for VPA and NAC-, or dimethylsulfoxide (DMSO) for 4-AP and 10H-PTZ.

### Cellular viability after treatment

Neuronal viability after treatments has been tested by 3-[4,5-dimethylthiazol-2-yl]-2,5 diphenyl tetrazolium bromide (MTT) assay (Table 1). To this aim, primary cortical neurons have been plated in 96-wells plates (3 × 10^4^ cells/cm^2^) and treated at div5 for 48 h. At div7 cells have been processed for the colorimetric assay following the manufacturer’s instructions [Cell Proliferation Kit I (MTT), Roche] and the absorbance has been quantified by a spectrophotometer (Tecan Infinite m nano) at 550 nm.

### Immunofluorescence

For immunofluorescence (IF) analysis, primary cortical neurons were seeded at the density of 8 × 10^4^ cells/cm^2^. 48 h after treatment, cells have been washed with Hanks’ Balanced Salt Solution (HBSS) and fixed with 4% paraformaldehyde (PFA) for 10 min as previously described^33^. Permeabilization and blocking of unspecific sites were performed in PBS/ 5% NDS/ 0.25% Triton-X100. Antibodies were incubated in PBS/ 5% NDS/ 0.1% Triton-X100. Primary antibodies used were: mouse anti-SMI31 (Biolegend, 1:1,000), rabbit anti-Synapsin (Sigma Aldrich, 1:1,000). Secondary antibodies used were: goat anti-mouse Alexa Fluor-488 (1:200) and goat anti-rabbit Alexa Fluor-594 (1:200). The slides were then coverslipped with anti-fade mounting medium Mowiol/Dabco.

### Morphological analysis

Morphometric analyses have been performed by Neurolucida software and the associated data analysis software NeuroExplorer (MBF Bioscience, Williston, VT): we evaluated soma size, neurite length and branching of 40 SMI31-positive neurons, (*n* = 10 per each experiment).

We also evaluated the signal intensity and distribution of synapsin labeling in 40 neurons (*n* = 10 per each experiment). Pictures at 40X magnification were taken by Eclipse E600 (Microfire Camera 2-Megapixel Color Imaging, 1600 × 1200): images were then identically thresholded for all the conditions and corrected total cell fluorescence (CTCF) was analyzed with ImageJ software.

### In vivo experiments in C. elegans

Nematodes have been grown and handled following standard procedures under uncrowded conditions on nematode growth medium (NGM) agar plates seeded with *Escherichia coli* strain OP50 ^60^. Strains used in this work are: NA1330 *gbIs4* [GBF109 *unc-25p::smn-1(RNAi);* GB301 *chs-2p::GFP*] III; NA1355 *gbIs4* III, *oxIs12* [*unc-47p::GFP; lin-15(+)*] X^27^.

NAC (Sigma Aldrich A7250) was dissolved in H_2_O and 10H-PTZ (Sigma Aldrich 88580) was dissolved in DMSO (Sigma Aldrich D-5879) and then added to molten NGM agar to obtain the desired final concentrations when poured into Petri dishes. Control plates contained the appropriate vehicle (H_2_O or DMSO) at similar volumes or final concentration. Animals have been treated from fertilization until young adult stage. Ten L4 larvae were left for 24 h to lay eggs and then removed from the plates, so that their F1 animals were scored at young adult stage. The phenotypes of dying MNs were scored by counting the number D-type MNs in the ventral cord acquiring autofluorescence as sign of apoptosis^27^. The death of neurons is detectable as the accumulation of apoptotic-related fluorescence in dying MNs, whose nature has been confirmed using cell death markers and genetic mutants (Gallotta et al., 2016). In wild-type worms there are no dying MNs. The degenerative phenotype was scored by counting the number of viable and therefore visible D-type MNs expressing GFP^61^. Animals were immobilized in 0.01% tetramisole hydrochloride (Sigma-Aldrich) on 4% agar pad and visualized using a Zeiss Axioskop microscope equipped with epifluorescence and DIC/Nomarski optics, and images were collected with an AxioCam digital camera. To discriminate dying MN fluorescence from endogenous autofluorescence, a Zeiss filter set 09 was used. This setting allowed the observation of intestinal cell autofluorescence in yellow and apoptotic fluorescence positive dying cells in green.

### Statistical analysis

In vitro data are shown as mean ± standard deviation (SD) of at least three independent cultures. In vivo data are shown as single dots for each animal adding mean ± standard error of the mean (SEM) of at least three independent experiments. Statistical analysis has been conducted by using: Unpaired Student t-test (morphometric analyses and synapsin signal intensity analysis); One-way ANOVA followed by Tukey’s multiple comparison test (MTT assay) One-way ANOVA followed by Dunnet Post Hoc Test (synapsin signal intensity analysis); unpaired non parametric Student t-test Mann-Whitney (quantification of the number of viable/visible MNs in *C. elegans*); unpaired non parametric One-way ANOVA Kruskal-Wallis Dunn’s multiple comparison test (quantification of the number of dying MNs in *C. elegans*). Statistical analysis has been performed using GraphPad Prism 8.0 software and GraphPad Prism version 10.1.2 for Windows (GraphPad Software, Boston, Massachusetts USA, http://www.graphpad.com). The number of experiments, the number of replicates within each experiment (n) and the statistical test are indicated in the figure legends. Differences were considered to be significant when *P* < 0.05.

## Supplementary Information

Below is the link to the electronic supplementary material.

Supplementary Material 1

## Data Availability

Raw data were generated at Neuroscience Institute Cavalieri Ottolenghi, Department of Neuroscience “Rita Levi Montalcini”, University of Turin, Turin, Italy and Institute of Biosciences and BioResources (IBBR), National Research Council (CNR), Naples, Italy. Derived data supporting the findings of this study are available from the corresponding authors and can be provided upon reasonable request.

## References

[1] d’Errico, P. et al. Selective vulnerability of spinal and cortical motor neuron subpopulations in delta7 SMA mice. *PLoS One*. **8**, e82654 (2013). 24324819 10.1371/journal.pone.0082654 PMC3855775

[2] Lefebvre, S. et al. Identification and characterization of a spinal muscular atrophy-determining gene. *Cell* **80**, 155–165 (1995). 7813012 10.1016/0092-8674(95)90460-3

[3] Burghes, A. H. M. & Beattie, C. E. Spinal muscular atrophy: why do low levels of survival motor neuron protein make motor neurons sick? *Nat. Rev. Neurosci.* **10**, 597–609 (2009). 19584893 10.1038/nrn2670 PMC2853768

[4] Finkel, R. S. et al. Nusinersen versus Sham control in Infantile-Onset spinal muscular atrophy. *N Engl. J. Med.* **377**, 1723–1732 (2017). 29091570 10.1056/NEJMoa1702752

[5] Mendell, J. R. et al. Single-Dose Gene-Replacement therapy for spinal muscular atrophy. *N Engl. J. Med.* **377**, 1713–1722 (2017). 29091557 10.1056/NEJMoa1706198

[6] Mercuri, E. et al. SUNFISH part 2: efficacy and safety of Risdiplam (RG7916) in patients with type 2 or Non-Ambulant type 3 spinal muscular atrophy (SMA) (1260). *Neurology* **94**, 1260 (2020).

[7] Moultrie, F., Chiverton, L., Hatami, I., Lilien, C. & Servais, L. Pushing the boundaries: future directions in the management of spinal muscular atrophy. *Trends Mol. Med.* **31**, 307–318 (2025). 39794178 10.1016/j.molmed.2024.12.006

[8] Reilly, A., Chehade, L., Kothary, R. & Curing, S. M. A. Are we there yet? *Gene Ther.* **30**, 8–17 (2023). 35614235 10.1038/s41434-022-00349-y

[9] Van Alstyne, M. et al. Gain of toxic function by long-term AAV9-mediated SMN overexpression in the sensorimotor circuit. *Nat. Neurosci.* **24**, 930–940 (2021). 33795885 10.1038/s41593-021-00827-3 PMC8254787

[10] Barbo, M. et al. Genetic variability in oxidative Stress, Inflammatory, and neurodevelopmental pathways: impact on the susceptibility and course of spinal muscular atrophy. *Cell. Mol. Neurobiol.* **44**, 71 (2024). 39463208 10.1007/s10571-024-01508-y PMC11513727

[11] Edinoff, A. N. et al. Phenothiazines and their evolving roles in clinical practice: A narrative review. *Health Psychol. Research* **10**, (2022). 10.52965/001c.38930 PMC9680852 36425230

[12] Zhang, W. et al. Phenothiazine confers neuroprotection via Dpp2/7 in high altitude traumatic brain injury mouse model. *High. Alt Med. Biol.*10.1089/ham.2024.0096 (2025). 40137938 10.1089/ham.2024.0096

[13] Hsu, K. C. et al. Synthesis and biological evaluation of phenothiazine derivative-containing hydroxamic acids as potent class II histone deacetylase inhibitors. *Eur. J. Med. Chem.* **219**, 113419 (2021). 33845233 10.1016/j.ejmech.2021.113419

[14] Ohlow, M. J. & Moosmann, B. Phenothiazine: the seven lives of pharmacology’s first lead structure. *Drug Discovery Today*. **16**, 119–131 (2011). 21237283 10.1016/j.drudis.2011.01.001

[15] Carocci, A. et al. Novel Phenothiazine/Donepezil-like hybrids endowed with antioxidant activity for a Multi-Target approach to the therapy of alzheimer’s disease. *Antioxid. (Basel)*. **11**, 1631 (2022). 10.3390/antiox11091631 PMC9495854 36139705

[16] Wang, X. X. et al. Synthesis and biological evaluation of selective histone deacetylase 6 inhibitors as multifunctional agents against alzheimer’s disease. *Eur. J. Med. Chem.* **225**, 113821 (2021). 34517222 10.1016/j.ejmech.2021.113821

[17] Keynes, R. G. et al. N10-carbonyl-substituted phenothiazines inhibiting lipid peroxidation and associated nitric oxide consumption powerfully protect brain tissue against oxidative stress. *Chem. Biol. Drug Des.* **94**, 1680–1693 (2019). 31127979 10.1111/cbdd.13572 PMC6790564

[18] Elshafay, A. et al. Efficacy and safety of valproic acid for spinal muscular atrophy: A systematic review and Meta-Analysis. *CNS Drugs*. **33**, 239–250 (2019). 30796634 10.1007/s40263-019-00606-6

[19] Marasco, L. E. et al. Counteracting chromatin effects of a splicing-correcting antisense oligonucleotide improves its therapeutic efficacy in spinal muscular atrophy. *Cell* **185**, (2022). 2057–2070.e15. 10.1016/j.cell.2022.04.031 PMC9555286 35688133

[20] Chiriboga, C. A. et al. Lack of effect on ambulation of dalfampridine-ER (4-AP) treatment in adult SMA patients. *Neuromuscul. Disord.* **30**, 693–700 (2020). 32788051 10.1016/j.nmd.2020.07.007

[21] Imlach, W. L. et al. SMN is required for sensory-motor circuit function in drosophila. *Cell* **151**, 427–439 (2012). 23063130 10.1016/j.cell.2012.09.011 PMC3475188

[22] Simon, C. M. et al. Chronic Pharmacological increase of neuronal activity improves Sensory-Motor dysfunction in spinal muscular atrophy mice. *J. Neurosci.* **41**, 376–389 (2021). 33219005 10.1523/JNEUROSCI.2142-20.2020 PMC7810663

[23] Wang, Z. B., Zhang, X. & Li, X. J. Recapitulation of spinal motor neuron-specific disease phenotypes in a human cell model of spinal muscular atrophy. *Cell. Res.* **23**, 378–393 (2013). 23208423 10.1038/cr.2012.166 PMC3587706

[24] Xu, C. C., Denton, K. R., Wang, Z. B., Zhang, X. & Li, X. J. Abnormal mitochondrial transport and morphology as early pathological changes in human models of spinal muscular atrophy. *Dis. Models Mech.* **9**, 39–49 (2016). 10.1242/dmm.021766 PMC4728333 26586529

[25] Stanga, S. et al. APP-dependent glial cell line-derived neurotrophic factor gene expression drives neuromuscular junction formation. *FASEB J.* **30**, 1696–1711 (2016). 26718890 10.1096/fj.15-278739

[26] Hoffmann, C. et al. Synapsin condensation controls synaptic vesicle sequestering and dynamics. *Nat. Commun.* **14**, 6730 (2023). 37872159 10.1038/s41467-023-42372-6 PMC10593750

[27] Gallotta, I. et al. Neuron-specific knock-down of SMN1 causes neuron degeneration and death through an apoptotic mechanism. *Hum. Mol. Genet.* **25**, 2564–2577 (2016). 27260405 10.1093/hmg/ddw119 PMC5181630

[28] Bowerman, M. et al. Therapeutic strategies for spinal muscular atrophy: SMN and beyond. *Dis. Model. Mech.* **10**, 943–954 (2017). 28768735 10.1242/dmm.030148 PMC5560066

[29] Pinzi, L., Bisi, N. & Rastelli, G. How drug repurposing can advance drug discovery: challenges and opportunities. *Front Drug Discov* **4**, (2024).

[30] Pushpakom, S. et al. Drug repurposing: progress, challenges and recommendations. *Nat. Rev. Drug Discov*. **18**, 41–58 (2019). 30310233 10.1038/nrd.2018.168

[31] Basak, S., Biswas, N., Gill, J. & Ashili, S. Spinal muscular atrophy: current medications and Re-purposed drugs. *Cell. Mol. Neurobiol.* **44**, 75 (2024). 39514016 10.1007/s10571-024-01511-3 PMC11549153

[32] Menduti, G., Rasà, D. M., Stanga, S. & Boido, M. Drug screening and drug repositioning as promising therapeutic approaches for spinal muscular atrophy treatment. *Front Pharmacol* **11**, (2020). 10.3389/fphar.2020.592234 PMC7689316 33281605

[33] Contino, S. et al. Presenilin-Deficient neurons and astrocytes display normal mitochondrial phenotypes. *Front. Neurosci.* **14**, 586108 (2020). 33551720 10.3389/fnins.2020.586108 PMC7862347

[34] Opsomer, R. et al. Amyloid Precursor Protein (APP) Controls the Expression of the Transcriptional Activator Neuronal PAS Domain Protein 4 (NPAS4) and Synaptic GABA Release. *eNeuro* 7, (2020). 10.1523/ENEURO.0322-19.2020 PMC7262005 32327470

[35] Groulx-Boivin, E. et al. Macrostructural brain abnormalities in spinal muscular atrophy. *Neurol. Genet.* **10**, e200193 (2024). 39308455 10.1212/NXG.0000000000200193 PMC11415185

[36] Hamilton, G. & Gillingwater, T. H. Spinal muscular atrophy: going beyond the motor neuron. *Trends Mol. Med.* **19**, 40–50 (2013). 23228902 10.1016/j.molmed.2012.11.002

[37] Yeo, C. J. J. & Darras, B. T. Overturning the paradigm of spinal muscular atrophy as just a motor neuron disease. *Pediatr. Neurol.* **109**, 12–19 (2020). 32409122 10.1016/j.pediatrneurol.2020.01.003

[38] Shababi, M., Lorson, C. L. & Rudnik-Schöneborn, S. S. Spinal muscular atrophy: a motor neuron disorder or a multi-organ disease? *J. Anat.* **224**, 15–28 (2014). 23876144 10.1111/joa.12083 PMC3867883

[39] Tapias, V., McCoy, J. L. & Greenamyre, J. T. Phenothiazine normalizes the NADH/NAD + ratio, maintains mitochondrial integrity and protects the nigrostriatal dopamine system in a chronic rotenone model of parkinson’s disease. *Redox Biol.* **24**, 101164 (2019). 30925294 10.1016/j.redox.2019.101164 PMC6440170

[40] Bykhovskaia, M. Synapsin regulation of vesicle organization and functional pools. *Semin. Cell Dev. Biol.* **22**, 387–392 (2011). 21827866 10.1016/j.semcdb.2011.07.003

[41] Chi, P., Greengard, P. & Ryan, T. A. Synaptic vesicle mobilization is regulated by distinct synapsin I phosphorylation pathways at different frequencies. *Neuron* **38**, 69–78 (2003). 12691665 10.1016/s0896-6273(03)00151-x

[42] Greengard, P., Valtorta, F., Czernik, A. J. & Benfenati, F. Synaptic vesicle phosphoproteins and regulation of synaptic function. *Science* **259**, 780–785 (1993). 8430330 10.1126/science.8430330

[43] Longhena, F. et al. An updated reappraisal of synapsins: structure, function and role in neurological and psychiatric disorders. *Neurosci. Biobehav Rev.* **130**, 33–60 (2021). 34407457 10.1016/j.neubiorev.2021.08.011

[44] Rocchi, A. et al. Autoantibodies to synapsin I sequestrate synapsin I and alter synaptic function. *Cell. Death Dis.* **10**, 1–16 (2019). 10.1038/s41419-019-2106-z PMC6856194 31727880

[45] Niceforo, A. et al. Altered cytoskeletal arrangement in induced pluripotent stem cells (iPSCs) and motor neurons from patients with riboflavin transporter deficiency. *Dis. Model. Mech.* **14** (dmm046391), dmm046391 (2021). 33468503 10.1242/dmm.046391 PMC7927654

[46] Briese, M. et al. Deletion of smn-1, the caenorhabditis elegans ortholog of the spinal muscular atrophy gene, results in locomotor dysfunction and reduced lifespan. *Hum. Mol. Genet.* **18**, 97–104 (2009). 18829666 10.1093/hmg/ddn320 PMC2644645

[47] Miguel-Aliaga, I. et al. The caenorhabditis elegans orthologue of the human gene responsible for spinal muscular atrophy is a maternal product critical for germline maturation and embryonic viability. *Hum. Mol. Genet.* **8**, 2133–2143 (1999). 10545592 10.1093/hmg/8.12.2133

[48] Sternberg, P. W. et al. WormBase 2024: status and transitioning to alliance infrastructure. *Genetics* **227**, iyae050 (2024). 38573366 10.1093/genetics/iyae050 PMC11075546

[49] Dimitriadi, M. et al. Conserved genes act as modifiers of invertebrate SMN loss of function defects. *PLoS Genet.* **6**, e1001172 (2010). 21124729 10.1371/journal.pgen.1001172 PMC2965752

[50] Chen, L. et al. Escape steering by cholecystokinin peptidergic signaling. *Cell. Rep.* **38**, 110330 (2022). 35139370 10.1016/j.celrep.2022.110330

[51] Di Giorgio, M. L. et al. WDR79/TCAB1 plays a conserved role in the control of locomotion and ameliorates phenotypic defects in SMA models. *Neurobiol. Dis.* **105**, 42–50 (2017). 28502804 10.1016/j.nbd.2017.05.005

[52] Hensel, N. et al. Impairment of the neurotrophic signaling hub B-Raf contributes to motoneuron degeneration in spinal muscular atrophy. *Proceedings of the National Academy of Sciences* 118, e2007785118 (2021). 10.1073/pnas.2007785118 PMC8106332 33931501

[53] Rademacher, S. et al. A single amino acid residue regulates PTEN-Binding and stability of the spinal muscular atrophy protein SMN. *Cells* **9**, 2405 (2020). 33153033 10.3390/cells9112405 PMC7692393

[54] de Cáceres, C. Automated screening of C. elegans neurodegeneration mutants enabled by microfluidics and image analysis algorithms. *Integr. Biology*. **10**, 539–548 (2018). 10.1039/c8ib00091c PMC6193809 30116818

[55] Rizzo, F. et al. Key role of SMN/SYNCRIP and RNA-Motif 7 in spinal muscular atrophy: RNA-Seq and motif analysis of human motor neurons. *Brain* **142**, 276–294 (2019). 30649277 10.1093/brain/awy330 PMC6351774

[56] Mazzarella, N. et al. Green Kiwifruit extracts protect motor neurons from death in a spinal muscular atrophy model in caenorhabditis elegans. *Food Sci. Nutr.* **7**, 2327–2335 (2019). 31367361 10.1002/fsn3.1078 PMC6657744

[57] Seo, J., Howell, M. D., Singh, N. N. & Singh, R. N. Spinal muscular atrophy: an update on therapeutic progress. *Biochim. Et Biophys. Acta (BBA) - Mol. Basis Disease*. **1832**, 2180–2190 (2013). 10.1016/j.bbadis.2013.08.005 PMC3825772 23994186

[58] Le, T. T. et al. SMNDelta7, the major product of the centromeric survival motor neuron (SMN2) gene, extends survival in mice with spinal muscular atrophy and associates with full-length SMN. *Hum. Mol. Genet.* **14**, 845–857 (2005). 15703193 10.1093/hmg/ddi078

[59] Hage, S. et al. Characterization of pterocarpus Erinaceus Kino extract and its gamma-secretase inhibitory properties. *J. Ethnopharmacol.* **163**, 192–202 (2015). 25639816 10.1016/j.jep.2015.01.028

[60] Brenner, S. The genetics of caenorhabditis elegans. *Genetics* **77**, 71–94 (1974). 4366476 10.1093/genetics/77.1.71 PMC1213120

[61] McIntire, S. L., Reimer, R. J., Schuske, K., Edwards, R. H. & Jorgensen, E. M. Identification and characterization of the vesicular GABA transporter. *Nature* **389**, 870–876 (1997). 9349821 10.1038/39908

