# Supplementary material for: 10H-phenothiazine exerts beneficial effects in spinal muscular atrophy in vitro and in vivo models
Source: Sci Rep. 2025 Dec 16;15:45757. doi: 10.1038/s41598-025-28547-9 (PMC12756266; doi:10.1038/s41598-025-28547-9)

**10H-PHENOTHIAZINE PROTECTS FROM NEURODEGENERATION IN SPINAL MUSCULAR ATROPHY *IN VITRO* AND *IN VIVO* MODELS**

**SUPPLEMENTAL FIGURE**

**Figure S1. Morphological analysis after NAC treatment using the highest concentration (5 mM).**

**A-C**. Morphological parameters of treated SMA cells measured using Neurolucida and NeuroExplorer software. Results are represented as mean ± SD of four independent experiments (n ≥ 10 neurons for each experiment). Student t-test: treated SMA vs vehicle no significant results.


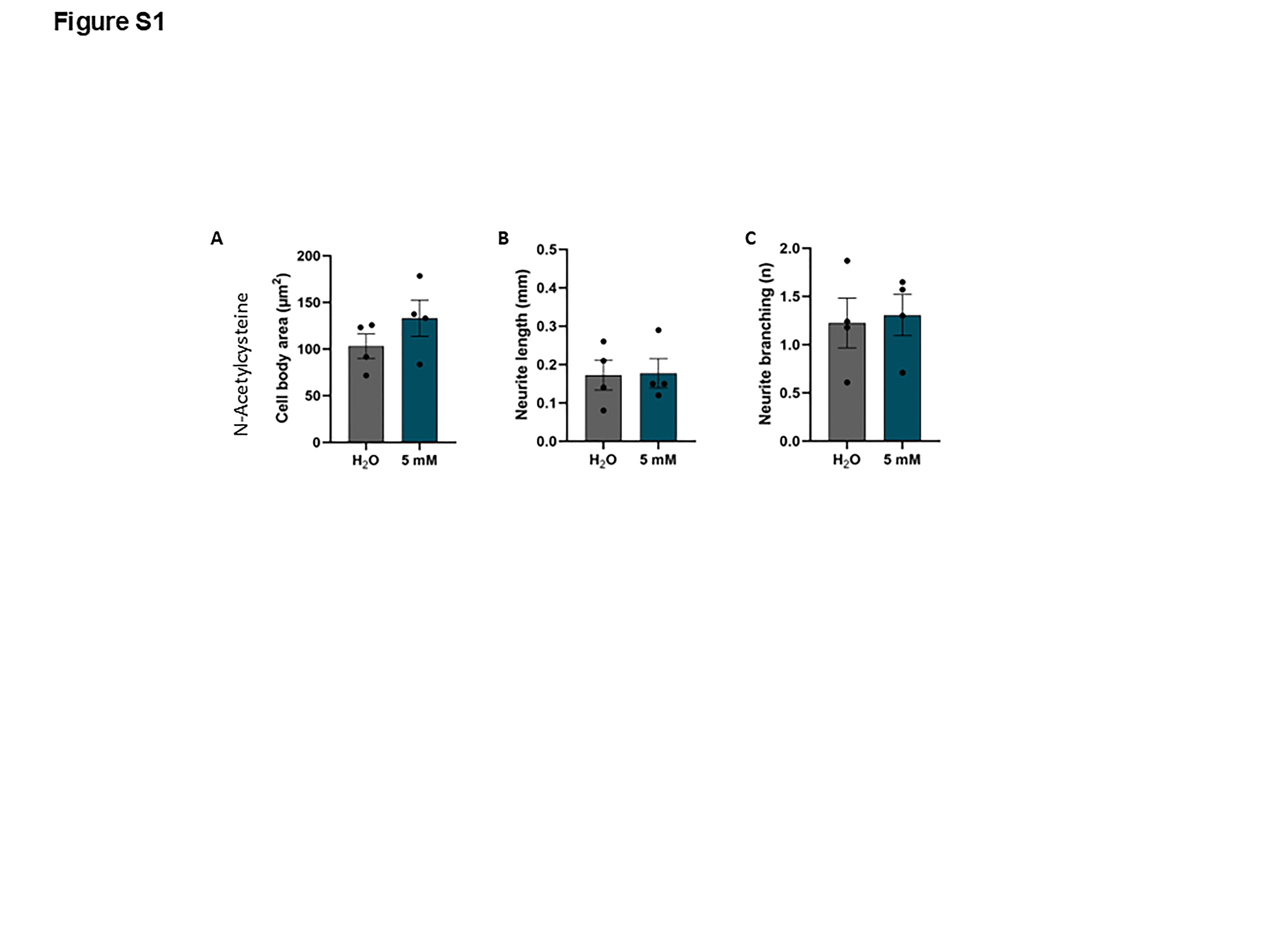

Supplement: Supplementary file 1 — Supplementary Material 1 [file 41598_2025_28547_MOESM1_ESM.docx]
